# Tetrahydroimidazo[4,5-*c*]pyridine-Based Inhibitors of *Porphyromonas gingivalis* Glutaminyl Cyclase

**DOI:** 10.3390/ph14121206

**Published:** 2021-11-23

**Authors:** Daniel Ramsbeck, Nadine Taudte, Nadine Jänckel, Stefanie Strich, Jens-Ulrich Rahfeld, Mirko Buchholz

**Affiliations:** 1 Fraunhofer Institute for Cell Therapy and Immunology IZI, Department of Drug Design and Target Validation MWT, Weinbergweg 22, 06120 Halle (Saale), Germany; stefanie.strich@gmail.com (S.S.); jens-ulrich.rahfeld@izi.fraunhofer.de (J.-U.R.); 2 PerioTrap Pharmaceuticals GmbH, Weinbergweg 22, 06120 Halle (Saale), Germany; nadine.taudte@periotrap.com (N.T.); nadine.jaenckel@periotrap.com (N.J.)

**Keywords:** PgQC, *Porphyromonas gingivalis*, periodontitis, glutaminyl cyclase

## Abstract

Periodontitis is a severe yet underestimated oral disease. Since it is linked to several systemic diseases, such as diabetes, artheriosclerosis, and even Alzheimer’s disease, growing interest in treating periodontitis has emerged recently. The major cause of periodontitis is a shift in the oral microbiome. A keystone pathogen that is associated with this shift is *Porphyromonas gingivalis*. Hence, targeting *P. gingivalis* came into focus of drug discovery for the development of novel antiinfective compounds. Among others, glutaminyl cyclases (QCs) of oral pathogens might be promising drug targets. Here, we report the discovery and structure–activity relationship of a novel class of *P. gingivalis* QC inhibitors according to a tetrahydroimidazo[4,5-*c*]pyridine scaffold. Some compounds exhibited activity in the lower nanomolar range and thus were further characterized with regard to their selectivity and toxicity.

## 1. Introduction

One of the most diverse microbiomes is found in the human oral cavity, where more than 700 different bacteria species have been identified [1,2]. They build up, together with Archaea, fungi, viruses, and protozoa, the “healthy” ecosystem, which is essential for maintaining both oral and systemic health. Disruption of this homeostasis, e.g., by lack of oral hygiene, genetic predisposition, tobacco and alcohol consumption, stress, aging, and immune disorders, can be the initial step for the development and progression of oral disease such as periodontitis, which is often accompanied by overgrowth of certain bacteria. In particular the Gram-negative, anaerobe bacteria *Porphyromonas gingivalis* has aroused special interest and is currently described as a keystone pathogen of periodontitis [3,4]. *P. gingivalis* expresses a variety of virulence factors such as proteinases (e.g., gingipains), LPS, capsule, fimbriae, outer membrane vesicles, and developed specific strategies to overcome the innate host immunity [5,6,7,8,9,10]. Once colonization of *P. gingivalis* has been manifested, it is able to induce a dysbiosis of the oral microbiota by immune subversion. An inadequate and enhanced host immune response results in inflammatory tissue destructions, which in turn provides nutrients for dysbiotic community. Thus, *P. gingivalis* supports the colonization of further oral pathogens such as *Treponema denticola* and *Tannerella forsythia*, both part of the “red” complex and markers of chronical periodontitis [11]. In short, periodontitis is a microbial-shift disease, where pathogenic bacteria become predominant and build up a polymicrobial dysbiotic community. This leads finally to degradation of tooth-supporting structures and, if not treated, subsequently to bone loss. Moreover, periodontitis is not only restricted to the oral cavity, but a number of studies also indicate an association of periodontitis with several chronic disorders such as type 2 diabetes mellitus, rheumatoid arthritis, cardiovascular disease, cancer, or Alzheimer’s disease [12,13,14,15,16,17].

Although periodontitis is the sixth most prevalent disease worldwide, with more than 740 million people affected [18], there is currently no long-term effective and selective treatment method available. The conventional, non-surgical treatment methods presently consist of supra- and subgingival scaling and root planning to remove the microbial dysbiotic biofilm often accompanied with the application of local or systemic antibiotics as an adjunct therapy [19,20]. As often described previously, the use of antibiotics has many drawbacks, such as the impairment or damage of the healthy microbiome and an increased risk of developing populations of antibiotic-resistant bacteria. Therefore, an alternative therapy that selectively prevents the formation of the dysbiotic biofilm would be desirable. One strategy compromises the specific intervention in the host immune and inflammatory system, such as complement inhibition by specific inhibitors [21,22] or a resolution of the excessive inflammatory response by resolvins [23].

*P. gingivalis* is considered to be a keystone pathogen of periodontitis that supports the growth of further pathogens and the shift of the oral microbiome towards a pathogenic biofilm. Thus, another treatment strategy could specifically target *P. gingivalis* through an inhibition of a physiological essential enzymes, e.g., glutaminyl cyclase (PgQC), by small molecules. PgQC belongs to the class of aminoacyl transferases and is presumably responsible for the cyclization of *N*-terminal glutamine residue of periplasmic, outer membrane-integrated, and extracellular proteins after their translocation into the periplasm [24,25]. The cyclization into pyroglutamate stabilizes virulence factors and protects them against proteolytic degradation. This protein modification and possibly other, yet unknown functions of PgQC seem to be essential for the physiological fitness of *P. gingivalis*. Hence, PgQC has been classified as an “essential” gene by transposon-mutagenesis experiments [26,27].

## 2. Results

### 2.1. Inhibitor Design

As a starting point for the inhibitor design, we recently solved the protein structure and identified PgQC as the first bacterial type II QC. According to this structure, the first compounds were developed, which exhibit significant effects on the growth of *P. gingivalis* [25].

**Figure 1:**
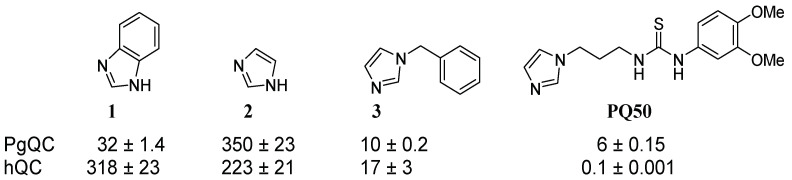
Comparison of inhibitory activities (K_i_-values in µM) of zinc-binding fragments and PQ50 against QC from *P. gingivalis* and human QC.

Several inhibitors of the homologous human glutaminyl cyclase (hQC), derived from either imidazole benzimidazole or other zinc-binding groups, have been reported [28,29,30,31,32,33,34,35,36,37,38,39,40]. The examination of the inhibitory potency of imidazole, imidazole derivatives, and benzimidazole as zinc binding fragments against PgQC revealed different activities from those observed in the human enzyme (Figure 1). While stronger inhibition was found for benzimidazole, the activity of *N*-benzylimidazole was in the same range, and the activity of imidazole was decreased compared to the inhibition of hQC. Surprisingly, the activity of PQ50, a potent hQC inhibitor, was decreased by a factor of 100 compared to hQC.

**Figure 2:**
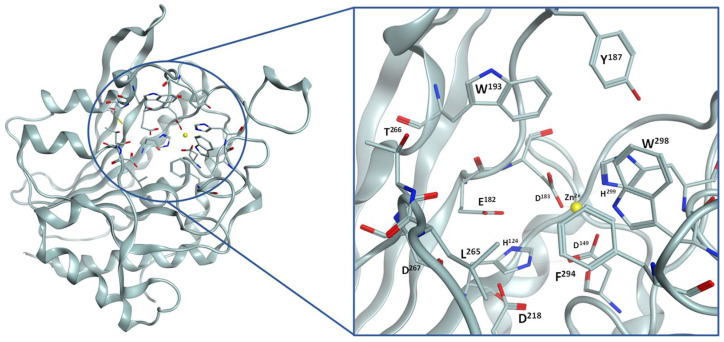
Left: Overall structure of PgQC (pdb: 6QQL); right: detailed view of the active site.

The structure of PgQC is depicted in Figure 2. The active site is characterized by the catalytic Zn-ion, which is coordinated by D^149^, D^183^, and H^299^. The bottom of the active site is formed by a cluster of acidic residues, i.e., D^218^, D^267^, and E^182^, and another histidine residue (H^124^). The entrance to this cavity harboring the catalytic Zn^2+^ is surrounded by rather lipophilic residues, i.e., Y^187^, W^193^, L^265^, F^294^, and W^298^.

Recently, we reported a first potent inhibitor of PgQC, according to imidazo[4,5-*b*]pyridine, i.e., an aza-analogue of benzimidazole **1 [25]**. Since there are also slight differences between the active sites of PgQC and hQC, we aimed at the development of further zinc-binding moieties that would enable a scaffold decoration different from the known QC inhibitors.

**Figure 3:**
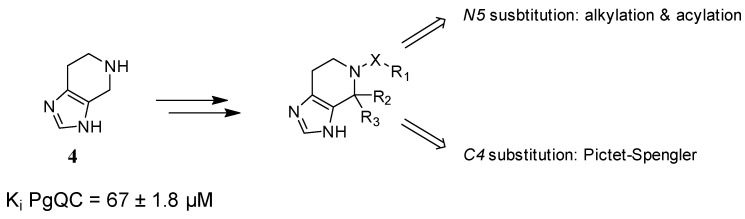
Possible decorations of the tetrahydroimidazopyridine scaffold.

We assumed that tetrahydroimidazo[4,5-*c*]pyridine might be a suitable scaffold with multiple connection points for further optimization by feasible chemistry. The unsubstituted tetrahydro-imidazo[4,5-*c*]pyridine exhibited an activity comparable to benzimidazole, thus indeed representing a good starting point for the development of PgQC inhibitors. Hence, we aimed at the introduction of further moieties (Figure 3), enabling interactions with the lipophilic rim at the entrance of the active site of PgQC.

### 2.2. Chemistry

**Figure 4:**
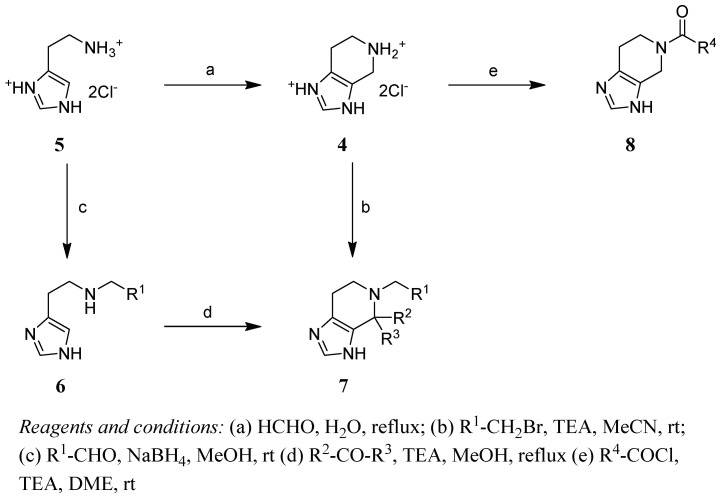
Synthesis of tetrahydroimidazo[4,5-*c*]pyridine derivatives.

The synthesis of the tetrahydro[4,5-*c*]pyridine core **4** was accomplished via Pictet–Spengler reaction of histamine hydrochloride and paraformaldehyde (Figure 4). Alkylation or acylation of the secondary amine yielding compounds **7a**,**b** and **8a**–**w** was achieved by treating **4** with different alkyl- or arylalkylbromides in acetonitrile or acid chlorides in dimethoxyethane at room temperature.

Compounds bearing an additional substituent at C_4_ (**7c**–**e**) were synthesized by reacting histamine with benzaldehyde, yielding intermediate **6**, followed by Pictet–Spengler cyclization using a suitable aldehyde or ketone under basic conditions.

### 2.3. SAR

Starting from **4**, the substitution at N_5_ of the tetrahydroimidazopyridine core with alkyl residues was investigated first (Table 1). The introduction of additional lipophilic moieties led to a generally improved activity compared to **4**. The introduction of a benzyl residue (**7a**) improved the activity by factor 60. Interestingly, the inhibitory activity was also significantly increased compared to the respective acyclic *N*-benzylhistamine derivative (**6a**), which is almost inactive against PgQC. This suggests a beneficial conformational preorganization due to the cyclization, which is mandatory for the potency of this scaffold. However, the introduction of an additional methylene group (**7b**) again led to a slightly decreased activity compared to **7a**. In accordance with these initial observations, the N5-benzyl moiety was kept constant and simple modifications at C_4_ were evaluated (**7c**–**e**). However, the introduction of substituents at this position led to decreased activities compared to **7a**, most likely due to steric hindrance, particularly in the case of the bulky phenyl residue (**7e**).

In addition to *N*-benzyl derivatives of tetrahydroimidazo[4,5-*c*]pyridine, also *N*-acyl-substituted compounds were synthesized and evaluated regarding their inhibitory activity. The evaluation of prototypic inhibitors **8a**–**d** revealed a superior activity of a N_5_-benzoyl moiety (**8a**) compared to phenyl-acetyl (**8b**), phenylpropionyl (**8c**), or cinnamoyl moieties (**8d**). This corroborates the finding of an optimal distance of one carbon between tetrahydroimidazopyridine core and the aryl moiety, which is also found within the N_5_-benzylderivatives **7a**,**b**. However, the introduction of an acyl moiety led to a further increase of activity compared to the respective alkyl derivative, e.g., **8a** compared to **7a**, or **8b** compared to **7b**. This suggests that there might be a further conformational fixation of the inhibitor. Moreover, due to the acylation, the basic center at N_5_ of the tetrahydroimidazopyridine is depleted. Thus, unfavorable electrostatic forces are reduced.

Since the *N*-acyl derivatives exhibited a slightly more potent inhibition of PgQC compared to the *N*-alkyl-substituted compounds, further modifications and SAR focused on the introduction of different benzoyl moieties (Table 2).

The introduction of electron-withdrawing fluorine (**8e**,**g**) led to a decreased activity compared to **8a**. However, a fluorine decoration in meta-position of the benzoyl moiety (**8f**) was tolerated and had no impact on the inhibitory activity. A comparable effect was observed for the introduction of chlorine. While a substitution in para position (**8h**) led to a decreased activity, compounds bearing a chlorine in meta position of the benzoyl residue (**8i**,**8j**) led to a slightly improved inhibitory activity. In particular, a 3,5-dichloro substitution (**8k**) led to an inhibitor with acceptable inhibitory potency against PgQC.

The introduction of electron-donating alkoxy residues (**8l**–**r**) reduced the activity compared to **8a** and the compounds bearing electron-withdrawing moieties, respectively, again with the exception of a substitution of the benzoyl residue in meta position (**8m**). However, the larger propyloxy moiety in meta-position (**8r**) led to decreased activity, probably indicating some steric hindrance within the active site. The combination of both a beneficial meta-methoxy and a meta-chloro substituent (**8t**) indeed led to an additive effect, revealing the most potent compound within this series.

**Figure 5:**
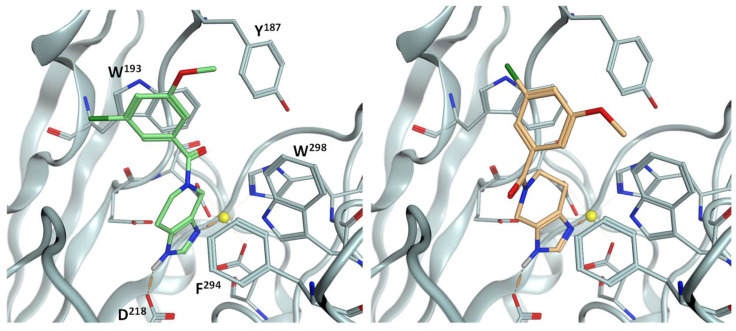
Putative binding mode of **8t**. Docking with GOLD, pdb: 6QQL. Left: 5-yl tautomer, solution ranked 2/20; right: 6-yl tautomer, solution ranked 1/20.

Docking experiments of **8t** were performed to gain insight into a possible binding mode within the active site of PgQC. The docking solutions of both possible imidazole-tautomers, i.e., the 5-acyl or 6-acyl substituted scaffolds, revealed quite similar binding modes (Figure 5). The imidazole is bound to the zinc ion and is further fixed within the active site via a hydrogen bond to D^218^ at the bottom of the cavity. The substituted benzoyl moiety of both tautomers forms hydrophobic contact to W^193^ and Y^187^, shaping the lipophilic entrance to the active site. In case of the 5-yl tautomer (Figure 5, left), a π-π interaction involving the chloro-methoxyaryl moiety and W^193^ could be formed.

However, no direct interaction of the amide C=O of the inhibitor with the protein could be observed. Hence, the slightly increased activity of the *N*-acyl-derived inhibitors compared to the *N*-alkyl substituted compounds cannot be explained by the binding mode revealed by the docking experiments. Moreover, it is not possible to deduce the bioactive tautomer of the inhibitor on the basis of these suggested binding modes.

The three most active compounds (**8k**,**t**,**w**) were further characterized regarding their inhibition of other glutaminyl cyclases from additional oral pathogens, i.e., *Prevotella intermedia* (PiQC) and *Tannerella forsythia* (TfQC), as well as the human isoenzyme (hQC).

As depicted in Table 3, compounds **8k**,**t**,**w** behave as broadspectrum inhibitors of type II glutaminyl cyclases, and no selectivity between the QCs from different species could be observed. However, the inhibition of QCs from other oral pathogens might be beneficial for the treatment of periodontitis since *T. forsythia* and *P. intermedia* are also involved in the development of the disease. In contrast, the inhibition of human QC could cause undesired side effects upon absorption of the inhibitors. Thus, this lack of selectivity might hamper the utilization of the reported inhibitors for periodontitis treatment. Further optimization of the compounds could solve this issue, either by tuning the selectivity or by a suitable decoration, omitting absorption to the systemic circulation, thus preventing inhibition of human QC in vivo. Nevertheless, they do not exhibit any cytotoxicity, rendering them as suitable lead compounds for further compound optimization studies.

## 3. Conclusions

The effective treatment of periodontitis is still an unmet medical need and due to its link to severe systemic diseases of growing interest for drug discovery. Bacterial type II glutaminyl cyclases recently emerged as potential drug targets for selectively combating the bacteria responsible for the development of periodontitis. Here, we aimed at the discovery of a novel scaffold for the development of novel inhibitors for these cyclases, in particular from *Porphyromonas gingivalis*. Within this study, a first insight of tetrahydro-imidazopyridines as PgQC inhibitors was gained. The decoration of the tetrahydroimidazopyridine scaffold led to inhibitors with activities in the lower nanomolar range. However, no selectivity for bacterial glutaminyl cyclases could be achieved, and the equipotent inhibition of the human isoenzyme was observed. Hence, this might hamper the further development of these inhibitors and could limit the suitability as potential periodontitis treatment. However, further optimization and decoration of the tetrahydropyridine-core might lead to compounds that target bacterial QCs more selectively. Thus, such compounds could indeed be useful agents for periodontitis treatment.

## 4. Materials and Methods

### 4.1. Chemistry

Starting materials and solvents were purchased from Aldrich, Activate Scientific, Alfa Aesar. The purity of the compounds was assessed by high-performance liquid chromatography (HPLC) and confirmed to be ≥95%. Analytical HPLC system was performed using a Merck–Hitachi device (model LaChrom) utilizing a Phenomenex Luna 5 μM C18(2) column (125 mm × 4.0 mm), with λ = 214 nm as the reporting wavelength. The compounds were analyzed using a gradient at a flow rate of 1 mL/min, whereby eluent (A) was acetonitrile and eluent (B) was water, both containing 0.04% (*v*/*v*) trifluoro acetic acid applying the following gradient: 0 min–15 min, 5–60% MeCN; 15 min–20 min, 60–95% MeCN; 20 min–30 min, 95 MeCN. The purities of all reported compounds were determined by the percentage of the peak area at 214 nm. ESI mass spectra were obtained with an Expression CMS spectrometer (Advion, Ithaca, NY 14850, USA). The high resolution ESI mass spectra were obtained from a LTQ Orbitrap XL (Thermo Fisher Scientific, Waltham, MA 02451, USA). The ^1^H NMR spectra were recorded at a DDR2 400 spectrometer (Agilent, Santa Clara, CA 95051, USA). DMSO-d6 was used as solvent unless otherwise specified. Chemical shifts are expressed as parts per million (ppm). The solvent was used as internal standard. Splitting patterns were designated as follows: s (singlet), d (doublet), dd (doublet of doublet), t (triplet), m (multiplet), and br (broad signal). Semipreparative HPLC was performed on a Prepstar device (Varian) equipped with a Phenomenex Luna 10 μM C18(2) column (250 mm × 21 mm). The compounds were eluted using the same solvent system as described above, applying a flow rate of 21 mL/min.

4,5,6,7-Tetrahydro-imidazo[4,5-*c*]pyridine (**4**)

Histamine dihydrochloride (**5**, 3.68 g; 20 mmol; 1 eq) and paraformaldehyde (1.20 g; 40 mmol; 2 eq) were dissolved in water (30 mL). The mixture was heated to reflux for 4 h. The volatiles were evaporated, and the residue was dried under vacuum. The compound was used without further purification. Yield: quantitative; ESI-MS *m*/*z*: 124.1 [M + H]^+^; HPLC: rt 1.25 min (>99%), ^1^H-NMR, 400 MHz, DMSO d6: δ 2.95–2.98, 3.42 (t, 2H, ^3^J = 5.9 Hz), 4.28 (s, 2H), 9.02 (s, 1H), 10.13 (br s, 2H), 14.83 (br s, 1H)

General Method *N*-Alkylation

A suspension of 4,5,6,7-tetrahydroimidazo[4,5-c]pyridine (**4**, 0.196 g; 1 mmol; 1 eq) in acetonitrile (10 mL) was treated with triethylamine (416 µL; 3 mmol; 3 eq), and the mixture was stirred at room temperature for 30 min. The respective halide (1 mmol; 1 eq) was added and the mixture was stirred at room temperature for further 12 h. The volatiles were evaporated, and the residue was taken up in water. The aqueous layer was extracted with EtOAc (3 × 20 mL). The combined organic layers were washed with brine, dried over Na_2_SO_4_, and evaporated. The product was purified by flash chromatography on silica using a CHCl_3_-MeOH gradient.

5-Benzyl-3,4,6,7-tetrahydroimidazo[4,5-*c*]pyridine (**7a**)

Yield: 38%; ESI-MS: *m*/*z* 214.1 [M + H]^+^; HPLC: 8.56 min (>99%); ^1^H-NMR, 400 MHz, DMSO d6: δ 2.56 (t, 2H, ^3^J = 5.5 Hz), 2.71 (t, 2H, ^3^J = 5.7 Hz), 3.34 (s, 2H), 3.67 (s, 2H), 7.24–7.30 (m, 1H), 7.33–7.36 (m, 4H), 7.40 (s, 1H), 11.65 (br s, 1H); HRMS: *m*/*z* 214.1332; calcd. for C_13_H_16_N_3_^+^: 214.1339.

5-(2-Phenylethyl)-3,4,6,7-tetrahydroimidazo[4,5-*c*]pyridine (**7b**)

Yield: 4%; ESI-MS: *m*/*z* 228.2 [M + H]^+^; HPLC: 5.41 min (>99%); ^1^H-NMR, 400 MHz, DMSO d6: δ 2.58 (t, 2H, ^3^J = 5.7 Hz), 2.77–2.86 (m, 6H), 3.53 (s, 2H), 7.16–7.21 (m, 1H), 7.24–7.31 (m, 4H), 7.46 (s, 1H). HRMS: *m*/*z* 228.1491; calcd. for C_14_H_18_N_3_^+^: 228.1495.

Synthesis of C4 substituted derivatives

Histamine (1.1 g; 10 mmol; 1 eq) was dissolved in methanol (30 mL). Benzaldehyde (1.02 mL; 10 mmol; 1 eq) was added, and the mixture was stirred at room temperature for 3 h. Sodium borohydride (567 mg; 15 mmol; 1.5 eq) was added in portions, and the reaction was stirred at room temperature for a further 3 h. The volatiles were evaporated, and the residue was taken up in water. The aqueous layer was extracted with EtOAc (3 × 50 mL). The combined organic layers were dried over Na_2_SO_4_ and evaporated. The product was used without further purification. Yield: 77%; ESI-MS: *m*/*z* 202.2 [M + H]^+^.

*N*-Benzylhistamine, obtained as described above (1 mmol; 1 eq), was dissolved in methanol (0.3–0.5 M). The respective aldehyde or ketone (1.2 mmol; 1.2 eq) and triethylamine (1 mmol; 1 eq) were added, and the mixture was heated to reflux overnight. The volatiles were evaporated, and the residue was taken up in water and a small amount of saturated aqueous NaHCO_3_. The aqueous layer was extracted with EtOAc (3 × 20 mL). The combined organic layers were washed with brine, dried over Na_2_SO_4_, and evaporated. The product was purified by flash chromatography on silica using a CHCl_3_-MeOH gradient.

5-Benzylspiro[6,7-dihydro-3H-imidazo[4,5-*c*]pyridine-4,3′-oxetane] (**7c**)

Yield: 52%; ESI-MS: *m*/*z* 256.2 [M + H]^+^; HPLC: 7.93 min (97.2%); ^1^H-NMR, 400 MHz, DMSO d6: δ 2.46 (t, 2H, ^3^J = 5.0 Hz), 2.67 (t, 2H, ^3^J = 5.7 Hz), 3.89 (s, 2H), 4.74–4.86 (m, 4H), 7.24–7.29 (m, 1H), 7.33–7.38 (m, 2H), 7.42–7.47 (m, 2H), 7.56 (s, 1H), 11.84 (br s, 1H).

5-Benzyl-4-methyl-3,4,6,7-tetrahydroimidazo[4,5-*c*]pyridine (**7d**)

Yield: 26%; ESI-MS: *m*/*z* 228.2 [M + H]^+^; HPLC: 3.79 min (95.3%); ^1^H-NMR, 400 MHz, DMSO d6: δ 1.29 (d, 3H, ^3^J = 6.1 Hz), 2.35–2.48 (m, 2H), 2.53–2.61 (m, 1H), 2.83–2.96 (m, 1H), 3.49–3.59 (m, 3H), 3.85 (d, 1H, J = 13.2 Hz), 7.22–7.27 (m, 1H), 7.29–7.38 (m, 4H), 7.42 (s, 1H), 11.66 (br s, 1H).

5-Benzyl-4-phenyl-3,4,6,7-tetrahydroimidazo[4,5-*c*]pyridine (**7e**)

Yield: 67%; ESI-MS: *m*/*z* 290.1 [M + H]^+^; HPLC: 9.68 min (>99%); ^1^H-NMR, 400 MHz, DMSO d6: δ 2.54–2.63 (m, 3H), 2.89–2.96 (m, 1H), 3.43 (d, 1H, J = 13.6 Hz), 3.66 (d, 1H, J = 13.6 Hz), 4.51 (s, 1H), 7.21–7.26 (m, 2H), 7.30–7.34 (m, 8H), 7.39 (s, 1H), 11.65 (br s, 1H).

General Method Acylation

A suspension of 4,5,6,7-tetrahydro-imidazo[4,5-c]pyridine (0.196 g; 1 mmol; 1 eq) in dimethoxyethane (10 mL) was treated with triethylamine (485 µL; 3.5 mmol; 3.5 eq), and the mixture was stirred at room temperature for 30 min. The solution was cooled to 0 °C, and the respective acyl halide (1 mmol; 1 eq) was added dropwise. After complete addition, the mixture was stirred at room temperature for 12 h. The volatiles were evaporated, and the residue was taken up in water. The aqueous layer was extracted with EtOAc (3 × 20 mL). The organic layer was washed with brine, dried over Na_2_SO_4_, and evaporated. The product was purified by flash chromatography on silica using a CHCl_3_-MeOH gradient.

Phenyl(3,4,6,7-tetrahydroimidazo[4,5-*c*]pyridin-5-yl)methanone (**8a**)

Yield: 15%; ESI-MS: *m*/*z* 228.1 [M + H]^+^; HPLC: 6.11 min (>99%); ^1^H-NMR, 400 MHz, DMSO d6: δ 2.62–2.71 (m, 2H), 3.60–3.85 (m, 2H), 4.49 (s, 2H), 7.41–7.50 (m, 6H), 11.72 (br s, 1H); HRMS: *m*/*z* 228.1127; calcd. for C_13_H_14_N_3_O^+^: 228.1131.

2-Phenyl-1-(3,4,6,7-tetrahydroimidazo[4,5-*c*]pyridin-5-yl)ethanone (**8b**)

Yield: 10%; ESI-MS: *m*/*z* 242.1 [M + H]^+^; HPLC: 7.04 min (95.9%); ^1^H-NMR, 400 MHz, DMSO d6: δ 3.71–3.86 (m, 4H), 4.48 (s, 2H), 7.18–7.35 (m, 5H), 7.44 (s, 1H), 11.64 (br s, 1H); HRMS: *m*/*z* 242.1282; calcd. for C_14_H_16_N_3_O^+^: 242.1288.

3-Phenyl-1-(3,4,6,7-tetrahydroimidazo[4,5-*c*]pyridin-5-yl)propan-1-one (**8c**)

Yield: 27%; ESI-MS: *m*/*z* 256.1 [M + H]^+^; HPLC: 7.65 min (94.5%); ^1^H-NMR, 400 MHz, DMSO d6: δ 2.56–2.62 (m, 2H), 2.68–2.75 (m, 2H), 2.84–2.92 (m, 2H), 3.65–3.81 (m, 2H), 4.44 (s, 2H), 7.16–7.21 (m, 1H), 7.24–7.30 (m, 4H), 7.44 (s, 1H), 11.63 (br s, 1H); HRMS: *m*/*z* 256.1439; calcd. for C_15_H_18_N_3_O^+^: 256.1444.

(E)-3-Phenyl-1-(3,4,6,7-tetrahydroimidazo[4,5-*c*]pyridin-5-yl)prop-2-en-1-one (**8d**)

Yield: 7%; ESI-MS: *m*/*z* 254.1 [M + H]^+^; HPLC: 8.56 min (97.3%); ^1^H-NMR, 400 MHz, DMSO d6: δ 2.68–2.72 (m, 2H), 3.92 (t, 2H, ^3^J = 5.5 Hz), 4.62 (s, 2H), 7.22–7.30 (m, 1H), 7.37–7,44 (m, 3H), 7.47–7.54 (m, 2H), 7.67–7.75 (m, 2H), 11.67 (br s, 1H); HRMS: *m*/*z* 254.1283; calcd. for C_15_H_16_N_3_O^+^: 254.1288.

(4-Fluorophenyl)-(3,4,6,7-tetrahydroimidazo[4,5-*c*]pyridin-5-yl)methanone (**8e**)

Yield: 18%; ESI-MS: *m*/*z* 246.1 [M + H]^+^; HPLC: 6.72 min (>99%); ^1^H-NMR, 400 MHz, DMSO d6: δ 2.57–2.72 (m, 2H), 3.43–3.70 (m, 1.4H), 3.76–3.99 (m, 0.6H), 4.25–4.66 (m, 2H), 7.25–7.32 (m, 2H), 7.45–7.57 (m, 3H), 11.91 (br s, 1H); HRMS: *m*/*z* 246.1030; calcd. for C_13_H_13_FN_3_O^+^: 246.1037.

(3-Fluorophenyl)-(3,4,6,7-tetrahydroimidazo[4,5-*c*]pyridin-5-yl)methanone (**8f**)

Yield: 40%; ESI-MS: *m*/*z* 245.1 [M + H]^+^; HPLC: 6.64 min (>99%); ^1^H-NMR, 400 MHz, DMSO d6: δ 2.59–2.73 (m, 2H); 3.53 (br s, 1H); 3.92 (br s, 1H); 4.33 (br s, 1H); 4.58 (s, 1H); 7.27–7.36 (m, 3H); 7.48–7.55 (m, 2H); 11.89 (br s, 1H).

(3,4,6,7-Tetrahydroimidazo[4,5-*c*]pyridin-5-yl)(3,4,5-trifluoro-phenyl)methanone (**8g**)

Yield: 35%; APCI-MS: *m*/*z* 282.0 [M + H]^+^; HPLC: 7.65 min (96.9%); ^1^H-NMR, 400 MHz, DMSO d6: δ 2.66 (br s, 2H); 3.49–3.59 (m, 1H); 3.89 (br s, 1H); 4.36 (s, 1H); 4.57 (s, 1H); 7.45–7.57 (m, 3H); 11.96 (br s, 1H).

(4-Chlorophenyl)-(3,4,6,7-tetrahydroimidazo[4,5-*c*]pyridin-5-yl)methanone (**8h**)

Yield: 34%; ESI-MS: *m*/*z* 262.1 [M + H]^+^; HPLC: 8.03 min (96.4%); ^1^H-NMR, 400 MHz, DMSO d6: δ 2.56–2.71 (m, 2H), 3.45–3.58 (m, 1H), 3.85–3.99 (m, 1H), 4.22–4.61 (m, 2H), 7.46–7.58 (m, 5H), 11.96 (br s, 1H). HRMS: *m*/*z* 262.0737; calcd. for C_13_H_13_ClN_3_O^+^: 262.0742.

(3-Chlorophenyl)-(3,4,6,7-tetrahydroimidazo[4,5-*c*]pyridin-5-yl)methanone (**8i**)

Yield: 32%; ESI-MS: *m*/*z* 262.1 [M + H]^+^; HPLC: 8.00 min (>99%); ^1^H-NMR, 400 MHz, DMSO d6: δ 2.59–2.73 (m, 2H), 3.49–3.59 (m, 1H), 3.87–3.97 (m, 1H), 4.29–4.39 (m, 1H), 4.58 (br s, 1H); 7.39–7.41 (m, 1H), 7.49–7.58 (m, 4H); 11.90 (br s, 1H).

(3,4-Dichlorophenyl)-(3,4,6,7-tetrahydroimidazo[4,5-*c*]pyridin-5-yl)methanone (**8j**)

Yield: 19%; ESI-MS: *m*/*z* 296.1 [M + H]^+^; HPLC: 9.52 min (>99%); ^1^H-NMR, 400 MHz, DMSO d6: δ 2.71–2.84 (m, 2H), 3.56–3.70 (m, 1.4H), 3.84–3.99 (m, 0.6H), 4.44–4.79 (m, 2H), 7.44–7.50 (m, 1H), 7.71–7.80 (m, 2H), 8.74–8.94 (m, 1H), 14.12 (br s, 1H). HRMS: *m*/*z* 296.0348; calcd. for C_13_H_12_Cl_2_N_3_O^+^: 296.0352.

(3,5-Dichlorophenyl)-(3,4,6,7-tetrahydroimidazo[4,5-*c*]pyridin-5-yl)methanone (**8k**)

Yield: 26%; APCI-MS: *m*/*z* 295.9 [M + H]^+^; HPLC: 9.23 min (98.3%); ^1^H-NMR, 400 MHz, DMSO d6: δ 2.61–2.68 (m, 2H); 3.53 (br s, 1H); 3.91 (br s, 1H); 4.33 (s, 1H); 4.58 (s, 1H); 7.52–7.56 (m, 3H); 7.74–7.75 (m, 1H); 11.95 (br s, 1H).

(4-Methoxyphenyl)-(3,4,6,7-tetrahydroimidazo[4,5-*c*]pyridin-5-yl)methanone (**8l**)

Yield: 4%; ESI-MS: *m*/*z* 258.1 [M + H]^+^; HPLC: 6.88 min (>99%); ^1^H-NMR, 400 MHz, DMSO d6: δ 2.79 (t, 2H, ^3^J = 5.5 Hz), 3.69–3.81 (m, 2H), 3.81 (s, 3H), 4.59–4.76 (m, 2H), 6.98–7.05 (m, 2H), 7.44–7.49 (m, 2H), 8.90 (s, 1H), 14.27 (br s, 1H). HRMS: *m*/*z* 258.1236; calcd. for C_14_H_16_N_3_O_2_^+^: 258.1237.

(3-Methoxyphenyl)-(3,4,6,7-tetrahydroimidazo[4,5-*c*]pyridin-5-yl)methanone (**8m**)

Yield: 5%; ESI-MS: *m*/*z* 258.1 [M + H]^+^; HPLC: 6.91 min (>99%); ^1^H-NMR, 400 MHz, DMSO d6: δ 2.69–2.83 (m, 2H), 3.61–3.68 (m, 1.4H), 3.79 (s, 3H), 3.85–4.01 (m, 0.6H), 4.42–4.81 (m, 2H), 6.99–7.04 (m, 2H), 7.07 (ddd, 1H, ^3^J = 8.3, ^4^J = 2.6, ^4^J = 0.9 Hz), 7.4 (t, 1H, ^3^J = 7.9 Hz), 8.75–8.96 (m, 1H), 14.22 (br s, 1H). HRMS: *m*/*z* 258.1230; calcd. for C_14_H_16_N_3_O_2_^+^: 258.1237.

(3,4-Dimethoxyphenyl)-(3,4,6,7-tetrahydroimidazo[4,5-*c*]pyridin-5-yl)methanone (**8n**)

ESI-MS: *m*/*z* 288.1 [M + H]^+^; HPLC (gradient 2): 5.89 min (%; doublepeak); ^1^H-NMR, 400 MHz, DMSO d6: δ 2.65–2.68 (m, 2H), 3.79–3.83 (m, 8H), 4.49 (br s, 2H); 7.01–7.03 (m, 3H), 7.53 (br s, 1H); 11.88 (br s, 1H).

(3,5-Dimethoxyphenyl)-(3,4,6,7-tetrahydroimidazo[4,5-*c*]pyridin-5-yl)methanone (**8o**)

Yield: 34%; ESI-MS: *m*/*z* 288.1 [M + H]^+^; HPLC (gradient 2): 7.71 min (92.9%); ^1^H-NMR, 400 MHz, DMSO d6: δ 2.58–2.68 (m, 2H); 3.54 (br s, 1H); 3.78 (s, 6H); 3.90 (br s, 1H); 4.34 (br s, 1H); 4.56 (br s, 1H); 6.54 (s, 2H); 6.58–6.59 (m, 1H); 7.48–7.53 (m, 1H); 11.88 (br s; 1H).

(2,3-Dihydrobenzo[b][1,4]dioxin-6-yl)(1,4,6,7-tetrahydro-5H-imidazo-[4,5-*c*]pyridin-5-yl)methanone (**8p**)

Yield: 33%; APCI-MS: *m*/*z* 286.0 [M + H]^+^; HPLC (gradient 2): 6.52 min (>99%); ^1^H-NMR, 400 MHz, DMSO d6: δ 2.63–2.66 (m, 2H); 3.58–3.91 (m, 2H); 4.28–4.30 (m, 4H); 4.47 (br s; 2H); 6.92–6.94 (m, 3H); 7.51 (s, 1H); 11.87 (br s, 1H).

Benzo[d][1,3]dioxol-5-yl(3,4,6,7-tetrahydroimidazo[4,5-*c*]pyridin-5-yl)methanone (**8q**)

Yield: 36%; ESI-MS: *m*/*z* 272.1 [M + H]^+^; HPLC (gradient 2): 6.53 min (>99%); ^1^H-NMR, 400 MHz, DMSO d6: δ 2.65 (br s, 2H); 3.52–3.90 (m, 2H); 4.47 (br s, 2H); 6.09 (s, 2H); 6.94–7.01 (m, 3H); 7.51 (s, 1H); 11.88 (br s, 1H).

(3-Propoxyphenyl)-(3,4,6,7-tetrahydroimidazo[4,5-*c*]pyridin-5-yl)methanone (**8r**)

Yield: 33%; APCI-MS: *m*/*z* 286.1 [M + H]^+^; HPLC: 9.23 min (86.6%); ^1^H-NMR, 400 MHz, DMSO d6: δ 0.98 (t, 3H, ^3^J = 7.3 Hz); 1.74 (sext, 2H, ^3^J = 6.8 Hz); 2.60–2.71 (m, 2H); 3.54 (br s, 1H); 3.86–3.98 (m, 3H); 4.34 (br s, 1H); 4.57 (br s, 1H); 6.94–7.08 (m, 3H); 7.35–7.39 (m, 1H); 7.50–7.56 (m, 1H); 11.94 (br s, 1H).

(4-Fluoro-3-methoxyphenyl)-(3,4,6,7-tetrahydroimidazo[4,5-*c*]pyridin-5-yl)methanone (**8s**)

Yield: 35%; APCI-MS: *m*/*z* 276.0 [M + H]^+^; HPLC: 7.10 min (>99%); ^1^H-NMR, 400 MHz, DMSO d6: δ 2.63–2.72 (m, 2H); 3.57 (br s, 1H); 3.88–3.95 (m, 4H); 4.38 (br s, 1H); 4.56 (br s, 1H); 7.00–7.04 (m, 1H); 7.23 (dd, 2H, ^4^J = 1.5 Hz, ^3^J = 8.3 Hz); 7.27–7.32 (m, 1H); 7.50–7.58 (m, 1H); 11.89 (br s; 1H).

(3-Chloro-5-methoxyphenyl)(1,4,6,7-tetrahydro-5H-imidazo[4,5-*c*]pyridin-5-yl)methanone (**8t**)

Yield: 36%; APCI-MS: *m*/*z* 292.1 [M + H]^+^; HPLC: 8.80 min (>99%); ^1^H-NMR, 400 MHz, DMSO d6: δ 2.62–2.71 (m, 2H); 3.53 (br s, 1H); 3.82 (s, 3H); 3.90 (br s, 1H); 4.32 (br s, 1H); 4.57 (br s, 1H); 6.95 (br s, 1H); 7.05 (s, 1H); 7.14 (s, 1H); 7.49–7.54 (m, 1H); 11.87 (br s, 1H).

[1,1′-Biphenyl]-4-yl(3,4,6,7-tetrahydroimidazo[4,5-*c*]pyridin-5-yl)methanone (**8u**)

Yield: 32%; ESI-MS: *m*/*z* 304.1 [M + H]^+^; HPLC: 10.61 min (>99%); ^1^H-NMR, 400 MHz, DMSO d6: δ 2.68 (s, 2H); 3.63 (br s, 1H); 3.94 (br s; 1H); 4.42–4.60 (m, 2H); 7.40–7.43 (m, 1H); 7.49–7.55 (m, 5H); 7.72–7.78 (m, 4H); 11.91 (br s, 1H).

[1,1′-Biphenyl]-3-yl-(3,4,6,7-tetrahydroimidazo[4,5-*c*]pyridin-5-yl)methanone (**8v**)

Yield: 33%; APCI-MS: *m*/*z* 304.1 [M + H]^+^; HPLC: 10.22 min (>99%); ^1^H-NMR, 400 MHz, DMSO d6: δ 2.60–2.76 (m, 2H); 3.61 (br s, 1H); 3.95 (br s, 1H); 4.41 (br s, 1H); 4.62 (s, 1H); 7.39–7.59 (m, 6H); 7.70–7.73 (m, 3H); 7.78–7.80 (m, 1H); 11.91 (br s, 1H).

Naphthalen-2-yl-(3,4,6,7-tetrahydroimidazo[4,5-*c*]pyridin-5-yl)methanone (**8w**)

Yield: 36%; ESI-MS: *m*/*z* 278.1 [M + H]^+^; HPLC: 9.20 min (>99%); ^1^H-NMR, 400 MHz, DMSO d6: δ 2.70 (br s, 2H); 3.61 (br s, 1H); 3.98 (br s, 1H); 4.43 (br s, 1H); 4.63 (br s, 1H); 7.44–7.64 (m, 4H); 7.98–8.04 (m, 4H); 11.89 (br s, 1H).

### 4.2. Docking

For the molecular docking of compound **8t**, the pdb file 6QQL (www.rcsb.org, accessed on 5 July 2021) was used as receptor. The docking procedure was set up in GOLD (v5.5). The active site was defined by the Zn^2+^ within a radius of 10 Å. For each compound, 20 docking runs were performed, with a set search efficacy of 100%, and ChemScore was used as the scoring function. The resulting solutions were imported and visualized in MOE (ChemicalComputingGroup, version 2019.0102). Only solutions with a plausible Zn binding were considered.

### 4.3. Inhibitor Assay

The enzymatic activity of recombinant QCs were evaluated using the fluorogenic substrate H-Gln-AMC as described previously [25]. For inhibitor testing, compounds were added with 1% (*v*/*v*) DMSO in the reaction mixture. Inhibitory constants were determined using concentrations of H-Gln-AMC varying from 1/4 K_M_ to 2 K_M_ and a final concentration of QC in a range between 20 and 50 nM. Progress curves were fitted to the general equation for competitive inhibition using GraFit software (Version 7, Erithacus software Ltd., Horley, UK).

## Figures and Tables

**Table 1:** Inhibition of PgQC by tetrahydroimidazo[4,5-*c*]pyridines with alkyl and acyl modifications.

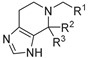	R^1^	R^2^	R^3^	K_i_ [µM]
**6a**	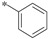	-		no inhibition @ 10 µM
**7a**	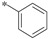	H	H	0.909 ± 0.005
**7b**	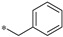	H	H	6.470 ± 0.400
**7c**	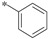	-CH_2_OCH_2_-		1.755 ± 0.145
**7d**	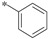	CH_3_	-	3.080 ± 0.040
**7e**	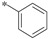	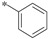	-	no inhibition @ 10 µM
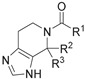				
**8a**	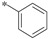	H	H	0.435 ± 0.015
**8b**	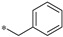	H	H	1.680 ± 0.080
**8c**	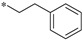	H	H	1.750 ± 0.060
**8d**	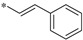	H	H	1.575 ± 0.025

**Table 2:** Inhibition of PgQC by *N*-benzoyltetrahydroimidazo[4,5-*c*]pyridines.

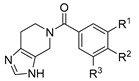	R^1^	R^2^	R^3^	K_i_ (µM)
**8e**	H	F	H	0.879 ± 0.008
**8f**	F	H	H	0.322 ± 0.006
**8g**	F	F	F	0.904 ± 0.007
**8h**	H	Cl	H	0.682 ± 0.025
**8i**	Cl	H	H	0.174 ± 0.014
**8j**	Cl	Cl	H	0.257 ± 0.016
**8k**	Cl	H	Cl	0.100 ± 0.006
**8l**	H	OCH_3_	H	0.833 ± 0.049
**8m**	OCH_3_	H	H	0.335 ± 0.006
**8n**	OCH_3_	OCH_3_	H	1.465 ± 0.304
**8o**	OCH_3_	H	OCH_3_	0.928 ± 0.021
**8p**	-OCH_2_CH_2_O-		H	1.335 ± 0.219
**8q**	-OCH_2_O-		H	0.560 ± 0.004
**8r**	OnPr	H	H	0.780 ± 0.024
**8s**	OCH_3_	F	H	1.240 ± 0.113
**8t**	OCH_3_	H	Cl	0.087 ± 0.006
**8u**	H	Ph	H	1.900 ± 0.071
**8v**	Ph	H	H	0.547 ± 0.041
**8w**	Ph		H	0.126 ± 0.009

**Table 3:** Inhibition of related glutaminyl cyclases from bacteria and human and cytotoxicity of selected inhibitors.

	8k	8t	8w
K_i_ (µM)			
PiQC	0.063 ± 0.007	0.096 ± 0.012	0.117 ± 0.002
TfQC	0.176 ± 0.003	0.221 ± 0.019	0.185 ± 0.010
hQC	0.387 ± 0.064	0.385 ± 0.096	0.132 ± 0.011
Cell viability @ 30 µM (%)			
SHSY-5Y	119	128	127
Hep-G2	92	96	94

## Data Availability

Data is contained within the article.
